# Causal effects of serum testosterone on septic shock mortality: a Mendelian randomization study

**DOI:** 10.1186/s13054-026-05860-x

**Published:** 2026-01-23

**Authors:** Nozomi Takahashi, Kyle R. Campbell, Taka-aki Nakada, Keith R. Walley

**Affiliations:** 1 Centre for Heart Lung Innovation, St. Paul’s Hospital, The University of British Columbia, 1081 Burrard Street, Vancouver, BC V6Z 1Y6 Canada; 2 Department of Emergency and Critical Care Medicine, Chiba University Graduate School of Medicine, 1-8-1 Inohana, Chuo, 260-8677 Chiba, Japan

**Keywords:** Sepsis, Septic shock, Lipids, Testosterone

## Abstract

**Background:**

Sex hormones, particularly testosterone, modulate immune function during critical illness, and patients with septic shock frequently exhibit hypotestosteronemia. However, the causal relationship between testosterone and outcomes remains unclear owing to the confounding effects of illness-related changes in hormone levels during acute illness.

**Methods:**

We investigated 469 patients with septic shock in multicenter ICUs using a testosterone polygenic score (PGS) derived from genome-wide association studies combined with two-sample Mendelian randomization to establish causal relationships independent of confounding factors. Cox proportional hazards regression was performed to assess the association with 28-day mortality. Additionally, we evaluated whether apolipoprotein C3 (ApoC3) levels modified the protective effects of testosterone using interaction models and the likelihood ratio test.

**Results:**

Higher genetically predicted testosterone levels were significantly associated with improved 28-day survival (adjusted hazard ratio [HR] 0.72 per 1-standard deviation increase in PGS; *P* = 0.024). This protective effect was more pronounced in men (HR, 0.66; *P* = 0.020) than in women (HR, 0.78; *P* = 0.37). Kaplan–Meier survival analysis revealed that the high testosterone PGS group had a 54.2% reduction in mortality hazard compared with the low testosterone PGS group (log-rank *P* = 0.007). Two-sample Mendelian randomization confirmed causality (inverse variance-weighted: β = − 2.79; *P* = 0.0042), with consistent results across complementary estimation methods. Notably, the testosterone-protective effect was significantly modified by ApoC3 levels (interaction, *P* = 0.041), with substantially stronger protective effects at higher ApoC3 concentrations. At high ApoC3 levels, the HR was 0.51 (95% confidence interval 0.31–0.85), suggesting that testosterone exerts a disproportionate benefit in the context of lipid dysmetabolism and inflammation.

**Conclusions:**

Genetically determined higher testosterone levels are causally associated with improved survival in patients with septic shock, particularly in men and in those with lipid dysmetabolism. These findings identify testosterone as a potential therapeutic target and highlight lipid metabolism as a key modifier of the protective effects of testosterone against septic shock, warranting the investigation of testosterone-based interventions in future clinical trials.

**Supplementary Information:**

The online version contains supplementary material available at 10.1186/s13054-026-05860-x.

## Background

Sepsis affects approximately 49 million individuals and causes 11 million deaths annually, accounting for one in five deaths worldwide [1, 2]. This life-threatening syndrome arises from a dysregulated host response to an infection, leading to organ dysfunction and failure. Although the implementation of sepsis alert systems has reduced the risk of mortality, hospital mortality remains high, with an overall rate of up to 40% [3]. These persistently high mortality rates underscore the urgent need for in-depth mechanistic insights and identification of novel therapeutic targets.

Sex hormones, particularly testosterone, have emerged as important modulators of immune function and sepsis outcomes [4]. Critically ill patients frequently exhibit hypotestosteronemia, and lower levels are associated with greater disease severity and mortality. Mechanistically, testosterone exerts complex, context-dependent effects on both innate and adaptive immunity; testosterone potentiates monocyte responses, leading to increased production of tumor necrosis factor, interleukin-6, and interleukin-15 [5]. In addition to its immunomodulatory effects, testosterone plays a crucial role in lipid metabolism by regulating triglyceride and cholesterol levels. Meta-analyses have shown that testosterone supplementation substantially reduces total cholesterol and triglycerides in hypogonadal men [6]. These hormone–lipid interactions are particularly relevant in critical illnesses, in which dysregulated lipid metabolism affects cellular energy utilization and inflammatory cascades [7].

However, testosterone levels measured during acute sepsis are confounded by illness-related suppression, medication effects, and rapid fluctuations, limiting causal inferences from observational studies [8, 9]. Genetic approaches using pre-illness exposure may overcome these limitations by providing clearer insights into causal relationships. Recent advances in genome-wide association studies (GWAS) have enabled the construction of robust polygenic scores (PGSs) for testosterone across diverse ancestries, capturing the genetic predisposition to lifelong hormone exposure [10]. The validity of these instruments has been demonstrated in Mendelian randomization (MR) frameworks, revealing causal relationships between testosterone and various metabolic and cardiovascular outcomes while minimizing confounding factors [11].

Therefore, in this study, we used testosterone PGS within an MR framework to investigate the causal relationship between genetically determined testosterone levels and sepsis outcomes. Furthermore, we explored interactions with lipid biomarkers to elucidate the potential lipid-mediated pathways linking testosterone to sepsis prognosis. By using this approach, we sought to obtain mechanistic insights into sex hormone regulation in critical illness and identify biomarkers for risk stratification in sepsis care.

## Methods

### Study design and definition

This multicenter observational study was conducted across multiple intensive care units (ICUs) in Japan to investigate patients with septic shock. We specifically targeted the septic shock population to minimize phenotypic heterogeneity and to focus on patients with profound circulatory and metabolic abnormalities, in whom the cardiovascular and immunomodulatory effects of testosterone were hypothesized to be most clinically relevant. Septic shock was defined according to the Third International Consensus Definitions for Sepsis and Septic Shock (Sepsis-3) criteria: the requirement of vasopressor therapy to maintain mean arterial pressure ≥ 65 mmHg and serum lactate levels > 2 mmol/L (18 mg/dL) despite adequate fluid resuscitation [12]. Genomic DNA extracted from the blood samples was genotyped using high-density single-nucleotide polymorphism (SNP) arrays, and a validated multi-ancestry PGS for serum testosterone was employed to predict each patient’s baseline testosterone levels. We then applied an MR framework to infer the potential causal relationships between genetically predicted testosterone levels and septic shock mortality. Additionally, we evaluated interactions between the testosterone PGS and concurrent lipid biomarker measurements to explore the lipid-mediated pathways influencing sepsis outcomes.

This study was approved by the Institutional Review Board of Chiba University Graduate School of Medicine and performed in accordance with the committee’s guidelines, including genetic analysis (Approval number 959). Written informed consent was obtained from all patients or their authorized representatives.

### Septic shock cohort and genotyping

We enrolled consecutive adult patients with septic shock admitted to ICUs between October 2012 and January 2022 across three academic hospitals and tertiary care centers in Chiba Prefecture, Japan. Among these patients, 687 met the Sepsis-3 definition of septic shock within 48 h of ICU admission. For the current analysis, we included patients with complete lipid profile measurements performed within 24 h of septic shock onset and available genotype data, following appropriate informed consent and quality control procedures for genetic analysis. Genomic DNA was extracted from buffy coat samples of discarded blood specimens collected within 24 h of septic shock onset. Principal component analysis was performed to assess the population structure, and only patients identified as having Japanese ancestry were included to ensure genetic homogeneity and reduce population stratification bias.

Genotyping was performed by using high-density SNP arrays. Detailed information regarding the SNP quality control procedures, imputation methods, and genotyping protocols has been described previously [13]. Standard quality control metrics, including call rates, Hardy-Weinberg equilibrium testing, and minor allele frequency thresholds, were applied to ensure high-quality genetic data for downstream PGS calculations.

### PGS for predicted serum testosterone levels

We employed a validated multi-ancestry PGS for serum testosterone, derived from data from more than 250,000 UK biobank data, to predict testosterone levels for each patient using 8,223 variants and their weighted effect sizes [14] (https://www.pgscatalog.org/, PGS000696). This score utilizes the BASIL algorithm and is constructed by finding the exact solution to L1-penalized multivariate regression (LASSO) on a large-scale dataset. This score was derived from the genetic combination of each individual’s parental alleles and was therefore unlikely to be influenced by unmeasured confounders. The septic shock cohort covered 7,750 (94.3%) variants. The PGS was calculated for each patient as the weighted sum of the effect allele dosages for 7,750 variants, multiplied by their corresponding effect weights (beta coefficients) obtained from the PGS Catalog (PGS000696). Patients were stratified into high and low testosterone groups based on the median PGS value of the cohort to ensure balanced sample sizes for comparative analysis, given the continuous nature of the genetic score and the absence of a pre-established clinical threshold. This value was used to determine the association between testosterone levels and mortality using a multivariate logistic regression model. Although the predicted testosterone levels were fundamentally determined by genetic predisposition, we included age, sex, body mass index, and corticosteroid use as covariates in multivariate analyses, as these factors reportedly influence both testosterone levels and septic shock prognosis [4, 15–17].

### Two-sample MR analysis

To strengthen the causal inference for the associations observed using the PGS approach, we performed a two-sample MR analysis. PGSs incorporate a broad spectrum of genetic variants, whereas MR applies more stringent selection criteria to instrumental variables, thereby providing a more robust framework for causal inferences.

Genetic instruments were constructed using summary statistics from the GWAS used to generate the testosterone PGS. Summary statistics were obtained from the GWAS Catalog (https://www.ebi.ac.uk/gwas/home, accession number GCST90019520). SNPs were selected as instrumental variables if they met the following criteria: genome-wide significance (*P* < 5 × 10⁻⁸) and independence with linkage disequilibrium *r²* < 0.001 within a 1 Mb clumping window. These criteria ensured the robustness and independence of the genetic instruments. Although the PGS was primarily derived from European ancestry data, its predictive validity has been confirmed in East Asian populations (*R*² > 0.9), supporting its transferability to our Japanese cohort. To minimize potential bias from population stratification and linkage disequilibrium differences across ancestries, we performed principal component analysis to ensure genetic homogeneity within the cohort and included principal components as covariates in our multivariate analyses. Of the selected SNPs, 53 were detectable in our septic shock cohort and were used as instrumental variables in the MR analysis (Supplementary material, Table E1). For the outcome, we used summary statistics for 28-day mortality in patients with septic shock, as previously reported [13].

### Statistical analyses

We analyzed the association between the calculated PGS, representing predicted baseline testosterone levels, and 28-day mortality in patients with septic shock using an MR framework (Supplementary material, Figure E1). SNPs constituting the PGS served as instrumental variables, with serum testosterone levels as the hypothetical exposure and 28-day mortality as the outcome [18]. This causal inference approach leverages the random allocation of genetic variants at conception to minimize the confounding and reverse causation inherent in observational studies of measured hormone levels [19]. Cox proportional hazards regression models were employed to assess the association between predicted testosterone levels and time to death within 28 days of septic shock onset. The associations between free of organ dysfunction during the first 28 days and testosterone PGS were calculated according to the Brussels criteria (Supplementary material, Table E2) and generated using a generalized linear model with Poisson regression.

The associations between predicted testosterone levels and lipid biomarkers measured during septic shock were examined using generalized linear models with appropriate link functions based on the distribution of individual biomarkers. For lipid markers demonstrating significant associations with the testosterone PGS, we constructed interaction models to determine whether these biomarkers modified the effect of predicted testosterone levels on septic shock outcomes. Likelihood ratio tests were performed to assess whether the inclusion of interaction terms significantly improved model fit, indicating effect modification of the prognostic impact of testosterone by specific lipid mediators.

For each instrumental variant, the effect of predicted serum testosterone levels on mortality was estimated using the inverse variance-weighted (IVW) method. This method assumes that all genetic variants used as instruments are valid, implying that they are associated with the exposure (serum testosterone levels) but not with any confounders or the outcome (mortality), except through the exposure. The IVW method combines the ratio estimates of each variant weighted by the inverse of their variance to provide an overall estimate of the causal effects. As some genetic variants may be invalid instruments owing to pleiotropy (where a single genetic variant influences multiple traits), additional estimation methods were employed. The weighted median method provides consistent estimates of causal effects, provided at least 50% of the weights come from valid instruments. This method is robust against the presence of invalid instruments and offers a more reliable estimate when some genetic variants exert pleiotropic effects.

To assess the robustness of the MR estimates and assess for potential violations of the MR assumptions, we performed multiple sensitivity analyses. MR-Egger regression was used to evaluate the horizontal pleiotropy by testing whether the intercept term deviated significantly from zero. We applied the MR-Pleiotropy Residual Sum and Outlier (MR-PRESSO) method to detect and correct horizontal pleiotropy by identifying outlier variants that may disproportionately influence the causal estimate. MR-PRESSO performed global and outlier tests; if significant outliers were detected, it provided outlier-corrected estimates after their removal. Additionally, we conducted a leave-one-out sensitivity analysis, systematically excluding each genetic variant in turn and recalculating the IVW estimate to assess whether any SNP exerted an undue influence on the overall MR result.

All statistical analyses were performed using R software (version 4.0 or later) (R Foundation for Statistical Computing, Vienna, Austria), and statistical significance was set at *P* < 0.05. In this study, the follow-up period was recorded to coincide with the primary outcome of 28 days, so long-term survival analysis was not performed. The analyses performed in this study followed the STROBE-MR statement.

## Results

A total of 469 patients with septic shock (median age, 69 years; 66.5% men; median Acute Physiology and Chronic Health Evaluation [APACHE] II score 30; Sequential Organ Failure Assessment [SOFA] score, 12) were included and stratified into high (*n* = 235) and low (*n* = 234) genetically predicted testosterone groups (Table 1). The two groups did not differ significantly in baseline demographics, comorbidities, or severity scores. The overall 28-day and in-hospital mortality rates were 11.3% and 30.1%, respectively.

**Table 1:** Clinical characteristics of the patients with septic shock

	All (*n* = 469)	High testosterone group (*n* = 235)	Low testosterone group (*n* = 234)	*P* value
Age, yr	69 [61–78]	70 [62–78]	69 [59–79]	0.25
Male sex, n (%)	312 (66.5)	148 (63.0)	164 (70.1)	0.13
BMI, kg/m^2^	22 [20–25]	23 [20–25]	22 [19–25]	0.32
APACHE II score^1)^	30 [24–36]	30 [24–36]	30 [23–37]	0.90
SOFA score^2)^	12 [9–15]	12 [9–15]	13 [10–17]	0.20
Comorbidity, n (%)				
Diabetes mellitus	125 (26.7)	61 (26.0)	64 (27.4)	0.81
Solid cancer	114 (24.3)	59 (25.1)	55 (23.5)	0.77
Dyslipidemia	55 (11.7)	33 (14.0)	22 (9.4)	0.16
Congestive heart failure	44 (9.4)	19 (8.1)	25 (10.7)	0.42
End stage renal failure	37 (7.9)	17 (7.2)	20 (8.5)	0.72
Chronic liver disease	34 (7.2)	14 (6.0)	20 (8.5)	0.37
Chronic pulmonary disease	26 (5.5)	14 (6.0)	12 (5.1)	0.85
Focus of infection, n (%)				
Lung	174 (37.1)	79 (33.6)	95 (40.6)	0.15
Abdominal	142 (30.3)	75 (31.9)	67 (28.6)	0.48
Skin and soft tissue	52 (11.1)	21 (8.9)	31 (13.2)	0.19
Genitourinary	43 (9.2)	26 (11.1)	17 (7.3)	0.20
Vital signs on Day1				
Body temperature, degrees	37.5 [36.6–38.5]	37.5 [36.5–38.5]	37.4 [36.4–38.4]	0.058
Heart rate, beats/min	110 [94–127]	111 [95–128]	110 [94–126]	0.68
Respiratory rate, /min	25 [20–30]	25 [19–31]	24 [19–29]	0.23
Mean arterial pressure, mmHg	73 [58–89]	77 [61–91]	71 [56–87]	0.062
Laboratory data on Day1				
Total bilirubin, mg/dL	1.0 [0.3–1.8]	1.0 [0.5–1.6]	1.1 [0.2–2.1]	0.29
Serum creatinine, mg/dL	1.5 [0.6–2.4]	1.5 [0.6–2.5]	1.4 [0.5–2.3]	0.50
White blood cell, 10^3^/µL	11.6 [5.9–17.3]	11.5 [5.8–17.2]	11.8 [6.1–17.5]	0.65
FDP^3)^, µg/mL	16.3 [2.3–30.3]	17.3 [3.5–31.0]	15.6 [1.3–30.0]	0.54
ICU duration, days	9 [5–19]	10 [5–19]	9 [5–18]	0.73

Data are median (interquartile range) for continuous variables

^1)^ APACHE, acute physiology and chronic health evaluation

^2)^ SOFA, sequential organ failure assessment

^3)^ FDP, fibrinogen/fibrin degradation products

### Association between testosterone PGS and septic shock prognosis

**Fig. 1:**
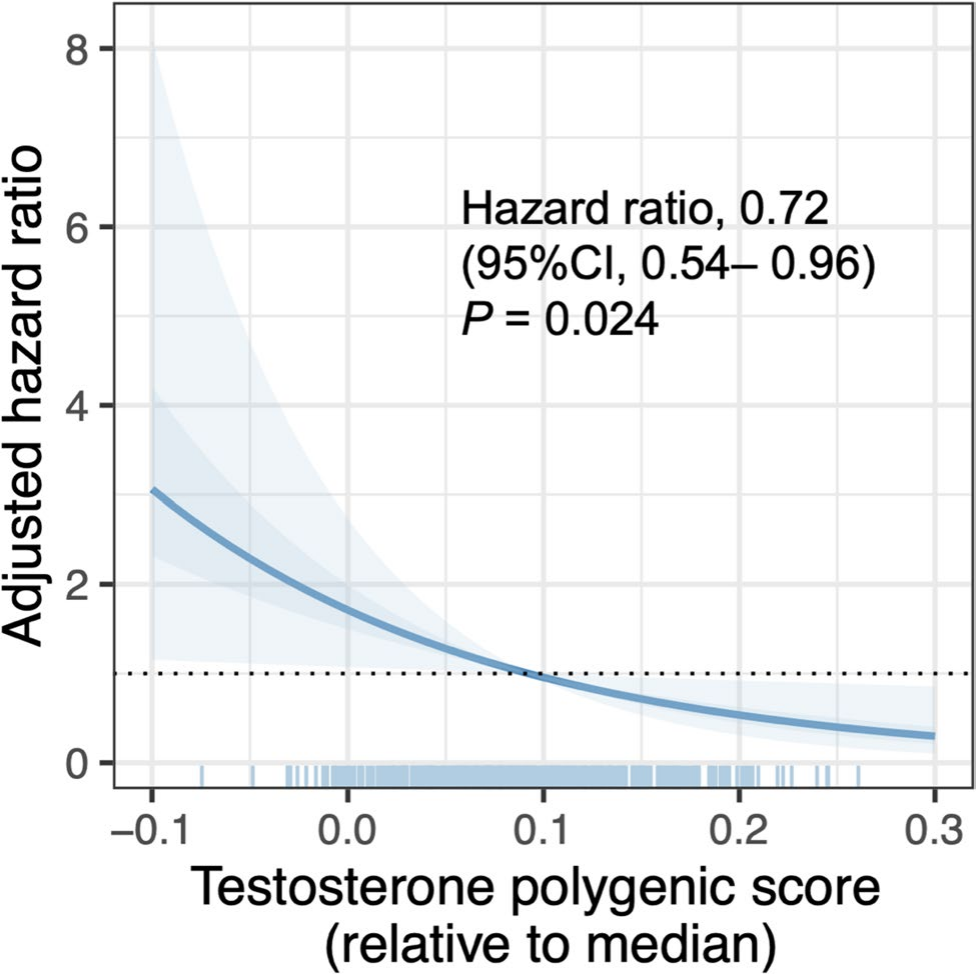
Association between testosterone polygenic score and 28-day mortality in patients with septic shock. The testosterone polygenic score for each individual was generated from weighted effect sizes from over 7,500 variants identified by GWAS analysis of serum testosterone levels. The hazard ratio for a 1-standard deviation (SD) increase in the testosterone polygenic score is 0.72 (95% confidence interval [CI], 0.54–0.96; *P* = 0.024). Light blue shading indicates 95% CI, and darker blue shading indicates standard error

**Fig. 2:**
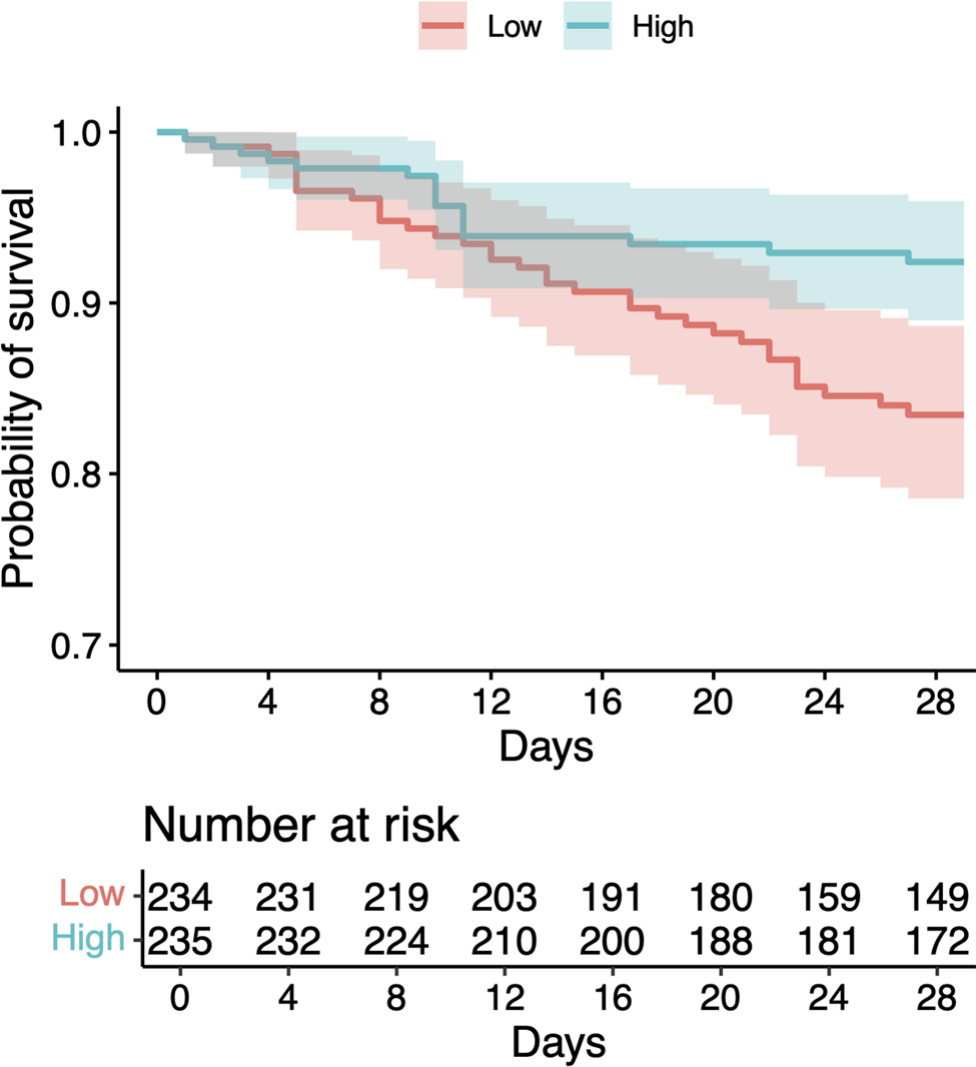
Kaplan–Meier survival analysis comparing patients with high versus low testosterone polygenic scores. The figure demonstrates significantly better overall survival in the high-scoring group (log-rank test, *P* = 0.007), showing a 54.2% reduction in mortality hazard

Cox proportional hazards regression analysis demonstrated that higher genetically predicted testosterone levels were significantly associated with improved survival in patients with septic shock. Higher testosterone PGSs were associated with reduced mortality risk, with an adjusted hazard ratio of 0.72 (95% confidence interval [CI], 0.54–0.96; *P* = 0.024) per 1-standard deviation (SD) increase in testosterone PGS relative to the median, suggesting the protective effect of genetically determined higher testosterone levels on septic shock outcomes (Fig. 1). Subgroup analysis according to sex revealed differential effects between male and female patients. Male patients exhibited a more pronounced protective effect, with an adjusted hazard ratio of 0.66 (95% CI, 0.46–0.94; *P* = 0.020), whereas the hazard ratio in female patients was 0.78 (95% CI, 0.46–1.34; *P* = 0.37). This finding indicated that the survival benefit associated with higher testosterone PGSs may be particularly relevant in male patients with septic shock. Kaplan-Meier survival analysis comparing patients with high versus low testosterone PGSs demonstrated significantly better overall survival in the high-score group (log-rank test, *P* = 0.007), showing a 54.2% reduction in the mortality hazard (Fig. 2).

### Association between testosterone PGS and organ dysfunction–free days

**Fig. 3:**
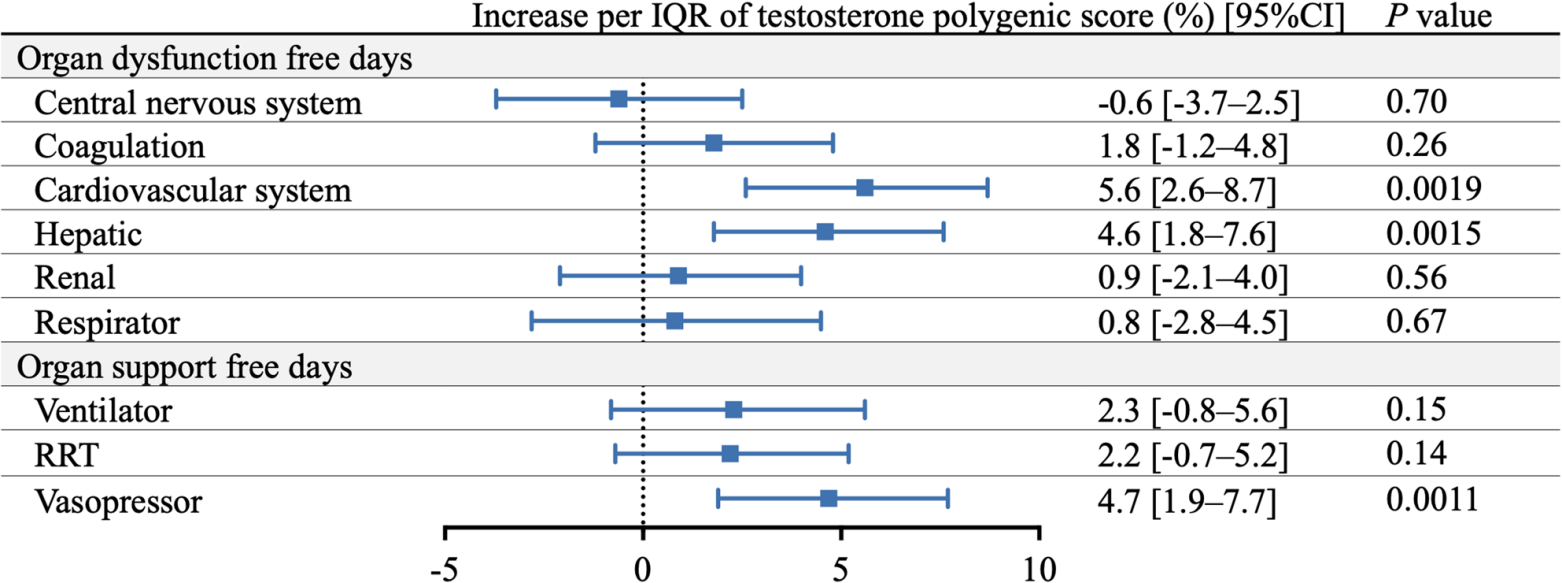
Association between days free of organ dysfunction and organ support and genetically predicted testosterone levels. IQR, interquartile range; CI, confidence interval; RRT, renal replacement therapy

Based on the Brussels criteria to quantify organ dysfunction, higher genetically predicted testosterone levels were significantly associated with increased days free of cardiovascular and hepatic dysfunction as well as vasopressor support during the first 28 days of septic shock. Each interquartile range increase in testosterone PGS was associated with a 5.6% increase in cardiovascular dysfunction–free days (95% CI, 2.6%–8.7%; *P* = 0.0019), a 4.6% increase in hepatic dysfunction–free days (95% CI, 1.8%–7.6%; *P* = 0.0015), and a 4.7% increase in vasopressor-free days (95% CI, 1.9%–7.7%; *P* = 0.0011). No significant association was observed with renal or respiratory dysfunction–free days (Fig. 3).

### Causal inference for serum testosterone levels and septic shock mortality

**Fig. 4:**
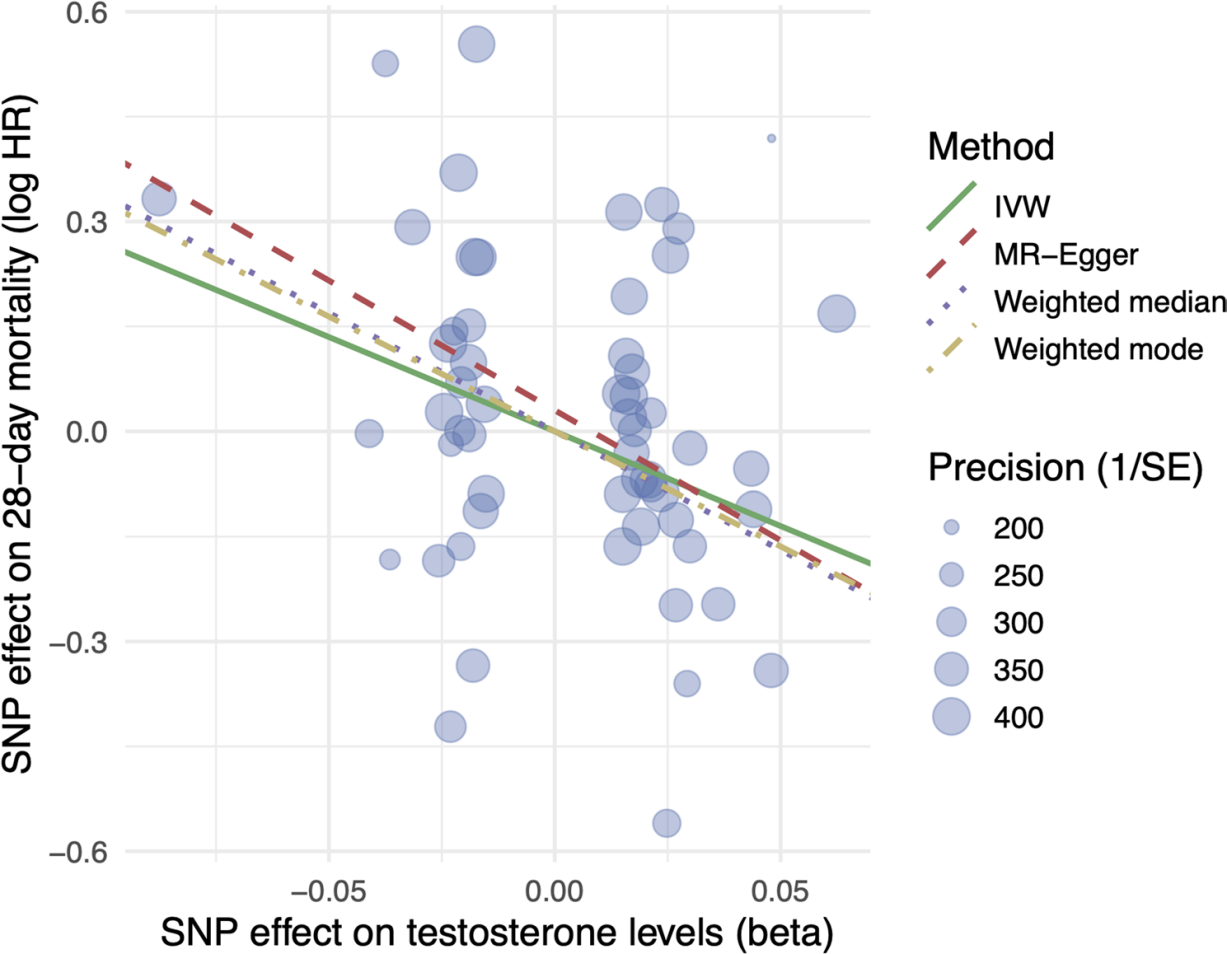
Two-sample Mendelian randomization for genetically predicted testosterone levels and 28-day mortality. Effect sizes are expressed as a change in testosterone level per standard deviation. MR, Mendelian randomization; IVW, inverse variance-weighted; SE, standard error; SNP, single-nucleotide polymorphism

Two-sample MR analysis identified a significant causal relationship between genetically predicted serum testosterone levels and 28-day mortality in patients with septic shock. The IVW method revealed a negative association (β = − 2.79, standard error [SE] = 0.94; *P* = 0.0042), indicating that higher genetically determined testosterone levels were causally linked to reduced mortality risk (Fig. 4). This finding was consistent across complementary MR methods, including the weighted median (β = − 3.38, SE = 1.45; *P* = 0.019) and weighted mode (β = − 3.30, SE = 1.54; *P* = 0.038) estimators, supporting the robustness of the association. The MR-Egger intercept (0.03; *P* = 0.63) suggested minimal horizontal pleiotropy and MR-PRESSO detected no outlier SNPs. Leave-one-out sensitivity analysis confirmed that no single SNP disproportionately influenced the overall effect estimate (Supplementary material, Figure E2). Collectively, these MR results reinforce the causal interpretation of the PGS findings and provide genetic evidence that elevated testosterone levels exert a protective causal effect on short-term mortality in septic shock.

### Association between testosterone PGS and lipid profiles in septic shock

To explore the potential metabolic pathways linking genetically predicted testosterone levels with septic shock outcomes, we examined the associations between testosterone PGSs and lipid profiles measured at the time of septic shock. Generalized linear models revealed significant associations of testosterone PGSs with triglycerides and apolipoprotein C3 (ApoC3), with ApoC3 demonstrating the strongest correlation (*P* = 0.013) (Supplementary material, Figure E3).

**Fig. 5:**
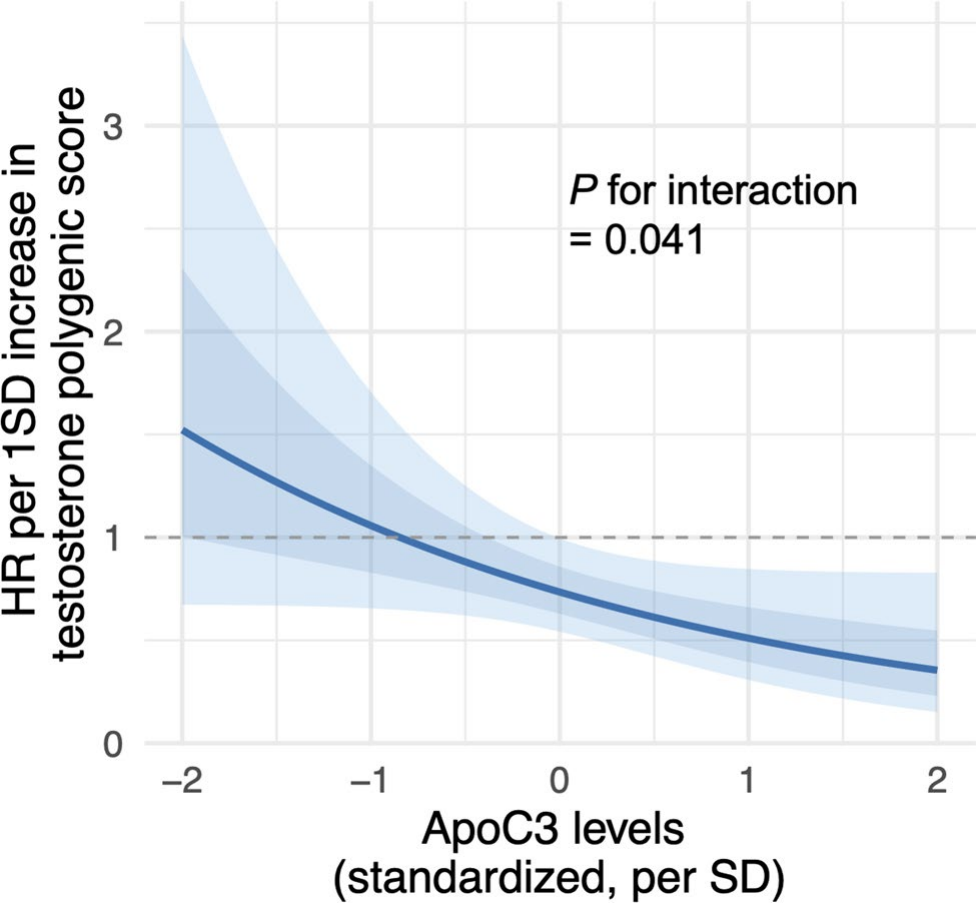
Change in HR by testosterone polygenic score at different levels of ApoC3 (standardized, SD units). Light blue shading indicates 95% confidence interval (CI) and darker blue shading indicates standard error. ApoC3, apolipoprotein C3; HR, hazard ratio; SD, standard deviation

Given this strong association, we evaluated whether ApoC3 levels modified the relationship between genetically predicted serum testosterone levels and 28-day mortality, using Cox regression analysis with an interaction term. The likelihood ratio test indicated that the inclusion of the interaction term significantly improved model fit (χ² = 4.18, df = 1; *P* = 0.041), demonstrating that the protective effect of genetically predicted testosterone on mortality varied according to ApoC3 levels. The relationship between the testosterone PGS and mortality risk showed a continuous dose-dependent interaction with ApoC3 levels (Fig. 5). At 1-SD below the mean ApoC3 level, each 1-SD increase in testosterone PGS was associated with a hazard ratio of 1.06 (95% CI, 0.66–1.71). Meanwhile, at mean ApoC3 levels, the hazard ratio was 0.73 (95% CI, 0.54–1.00), whereas at 1-SD above the mean ApoC3 level, the hazard ratio decreased to 0.51 (95% CI, 0.31–0.85). These findings indicated that the protective effect of genetically predicted testosterone on septic shock mortality increases progressively with increasing ApoC3 levels, suggesting an interplay between testosterone and lipid metabolism in determining septic shock outcomes.

## Discussion

In this study involving 469 patients with septic shock, we found that higher genetically predicted serum testosterone levels were significantly associated with improved 28-day survival, as demonstrated by both PGS analysis and two-sample MR analysis. This protective effect was more pronounced in men and was independent of baseline organ dysfunction. Notably, the testosterone–mortality association was significantly modified by ApoC3 levels, suggesting the mediating role of lipid metabolism in the relationship between testosterone levels and septic shock outcomes.

Our finding that genetically predicted higher testosterone levels were associated with improved survival in patients with septic shock represents an important contribution to our understanding of the role of sex hormones in the pathophysiology of sepsis. This result contrasts with the conventional understanding based on observational studies, which have consistently demonstrated that male patients with septic shock experience worse outcomes than female patients [20, 21]. The apparent paradox between poor outcomes in men and the protective effects of testosterone in our study can be reconciled by considering the temporal and physiological dynamics of testosterone levels during critical illness. In healthy individuals and under normal conditions, testosterone exerts immunosuppressive effects on both innate and adaptive immunity, as manifested by reductions in immunoglobulin production, cytokine synthesis, and lymphocyte proliferation [20]. However, the pathophysiology shifts dramatically during septic shock. The initial hyperinflammatory phase is characterized by uncontrolled cytokine release and excessive proinflammatory responses, including elevated levels of tumor necrosis factor-α and interleukin-6 [21–23]. In this context, the immunosuppressive properties of testosterone may be beneficial because testosterone modulates the harmful cascade of excessive inflammation, which drives organ dysfunction and mortality. Furthermore, testosterone possesses vasodilatory properties and enhances cardiovascular stability via multiple mechanisms. Experimental evidence indicated that exogenous testosterone administration prevents mortality in endotoxemic animal models, particularly when nitric oxide production is impaired [24]. During septic shock, which hallmark features include distributive shock and vascular dysfunction, the cardiovascular protective effects of testosterone may be particularly valuable for maintaining perfusion and preventing progressive organ failure.

The sex-stratified analysis in the present study further supports this hypothesis. The stronger protective association in men suggests that the biological effects of testosterone are more pertinent in the male physiological context, where endogenous testosterone levels are naturally higher. Our MR analysis established causality independent of confounding factors, thereby reinforcing the concept that genetically determined higher testosterone levels independently contribute to improved survival in septic shock and supporting a causal rather than an observational relationship. These findings highlight that the disadvantage observed in men during sepsis may not be attributable to the testosterone itself but rather to the dramatic depletion of testosterone that occurs during critical illness combined with other male-specific immune vulnerabilities, such as reduced type I interferon responses and heightened proinflammatory activation [5]. Future therapeutic strategies targeting testosterone restoration or optimization during septic shock warrant investigation as potential avenues for improving patient outcomes, particularly in male patients.

We found that the protective effect of testosterone on septic shock mortality was significantly modified by ApoC3 levels, with stronger protective effects observed at higher ApoC3 concentrations. This novel interaction suggests that testosterone exerts potent anti-inflammatory effects against lipid-driven inflammation [25, 26]. Testosterone modulates lipid metabolism through multiple pathways, including the upregulation of scavenger receptor B1 and hepatic lipase, thereby promoting favorable lipid remodeling and reducing proinflammatory lipoprotein abundance [27]. In patients with sepsis exhibiting elevated ApoC3 levels, characterized by pronounced lipid dysmetabolism and heightened NLRP3-mediated inflammation, testosterone may exert disproportionate clinical benefits by tempering ApoC3-driven inflammasome activation and related organ dysfunction. Conversely, at lower ApoC3 levels, the inflammatory landscape was dominated by mediators that were less responsive to the protective mechanisms of testosterone. These findings highlight the importance of considering the lipid metabolic phenotype when evaluating the effects of testosterone on septic shock, as the clinical relevance of sex hormone interventions may vary substantially depending on the underlying inflammatory and lipid profiles. Taken together, testosterone replacement or supplementation has the potential as adjunctive therapeutic strategy in genetically susceptible patients with sepsis and lipid dysmetabolism, warranting further investigations.

This study has some limitations that warrant discussion. First, serum testosterone levels were not directly measured at the time of septic shock. However, the genetic approach avoids the confounding from acute illness-induced suppression and ICU medications. The genetically predicted score reflects pre-morbid baseline status, and we adjusted for age, sex, BMI, and corticosteroid use in multivariate analyses. While this approach does not measure acute levels, it provides more robust causal inference than observational studies of measured hormones during critical illness. Second, PGS analysis may be subject to unmeasured confounding factors; nevertheless, we employed a two-sample MR to strengthen the causal inference and address this limitation. Third, we observed a discrepancy between the low 28-day mortality and higher in-hospital mortality. This reflects the specific healthcare context in Japan, where universal access to intensive care and a cultural reluctance to withdraw life-sustaining treatment often prolong the clinical course of non-survivors beyond 28 days [28, 29]. To validate whether our findings are generalizable across diverse healthcare systems and end-of-life care practices, international multicenter studies with standardized mortality endpoints are warranted. Additionally, the requirement for informed consent for genetic analysis may have introduced a survival bias by excluding patients with fulminant courses who died before enrollment, potentially contributing to the lower observed 28-day mortality despite high admission severity scores. Finally, the cross-sectional nature of lipid measurements, with ApoC3 assessed at a single time point during sepsis, limits the interpretation of temporal dynamics between lipid metabolism and the testosterone-mortality relationship. Prospective studies using serial testosterone and lipid measurements are required to elucidate the dynamic interplay between these factors and septic shock outcomes.

## Conclusions

This study provides genetic and causal evidence that higher serum testosterone levels were associated with improved survival in patients with septic shock, with a protective effect that was particularly pronounced in men and was substantially amplified by lipid dysmetabolism. These findings indicate that testosterone replacement or supplementation may represent a novel therapeutic strategy for improving septic shock outcomes, particularly in genetically susceptible individuals with dysregulated lipid metabolism. Further interventional studies are warranted to evaluate whether testosterone modulation can be therapeutically exploited to reduce septic shock-related mortality.

## Supplementary Information

Below is the link to the electronic supplementary material.

Supplementary Material 1

## Data Availability

The datasets used and analyzed in the current study are available from the corresponding author upon reasonable request and ethics approval.

## Abbreviations

ICU: Intensive care unit
SOFA: Sequential Organ Failure Assessment
SNP: Single-nucleotide polymorphism
APACHE: Acute Physiology and Chronic Health Evaluation
GWAS: Genome-wide association study
CI: Confidence interval
HR: Hazard ratio
MR: Mendelian randomization
IVW: Inverse variance-weighted
SE: Standard error
PGS: Polygenic score
MR-PRESSO: MR-Pleiotropy Residual Sum and Outlier
SD: Standard deviation
