# Supplementary material for: Causal effects of serum testosterone on septic shock mortality: a Mendelian randomization study
Source: Crit Care. 2026 Jan 23;30:84. doi: 10.1186/s13054-026-05860-x (PMC12910909; doi:10.1186/s13054-026-05860-x)

Supplementary material

Causal Effects of Serum Testosterone on Septic Shock Mortality: A Mendelian Randomization Study

Authors: Nozomi Takahashi, Kyle R. Campbell, Taka-aki Nakada, Keith R. Walley

Table contents:

Genetic analysis for septic shock cohort.

1. Septic Shock Cohort
2. Genotype Quality Control and Population Structure Analysis
3. DNA Extraction and Genotyping
4. Two-Sample Mendelian Randomization analysis

Tables:

Table E1. Genome-wide significant variants for serum testosterone levels
Table E2. Brussels organ dysfunction definitions

Figures:

Figure E1. Adjusted models for genetically predicted testosterone levels and 28-day mortality
Figure E2. The effect of each SNP on Mendelian randomization analysis
Figure E3. Association of genetically predicted testosterone levels with lipoproteins measured during septic shock

Genetic analysis for septic shock cohort

1. Septic Shock Cohort

In this multicenter observational study, we screened all patients admitted to intensive care units (ICUs) across Chiba Prefecture, Japan, from October 2012 to January 2022. Eligible participants were adults who met Sepsis-3 criteria for septic shock, defined as an acute increase in the Sequential Organ Failure Assessment (SOFA) score of two or more points due to infection, persistent hypotension requiring vasopressor therapy to maintain mean arterial pressure ≥65 mmHg despite adequate fluid resuscitation, and a serum lactate level greater than 2 mmol/L within 48 hours of ICU admission. Inclusion further required availability of plasma samples for both lipoprotein measurement and genotyping analysis.

A total of 469 patients met all criteria and were included in the final analysis. All subjects were confirmed to be of Japanese ancestry by population structure analysis to ensure genetic homogeneity. The study protocol was approved by the Institutional Review Board of Chiba University (approval number 959) and conducted in accordance with institutional guidelines, including genetic analysis procedures. Written informed consent was obtained from all participants or their legally authorized representatives before enrollment.

2. Genotype Quality Control and Population Structure Analysis

Genotyping quality control was performed using stringent criteria to ensure data integrity. The Call Rate (CR) was calculated for each individual sample and defined as the percentage of successfully genotyped single nucleotide polymorphisms (SNPs) on the microarray. Samples with CR values below 0.98 were excluded from further analysis, with this threshold applied uniformly across the cohort.

To assess ancestral relatedness and exclude potential relatives, identity-by-descent (IBD) analysis was performed using SNP data. Pairs of samples exhibiting identity-by-descent expectation values (PIHAT) exceeding 0.1875 were flagged, and one sample from each related pair was excluded to ensure statistical independence. Additionally, genomic quality control included checks for sample mishandling, DNA contamination, and consistency between reported and genotypic sex.

Population structure was evaluated using principal component analysis (PCA) on genome-wide SNP data, comparing the study population against reference ancestry data from 2,504 individuals representing 25 ancestral populations from the 1000 Genomes Project. Study samples were visually stratified based on scatter plots of the first and second principal components to confirm Japanese ancestry. All SNP data were harmonized to a common reference build, and palindromic variants (A/T and G/C) were removed prior to analysis to prevent strand-assignment errors.

<u>Structured analysis of populations using principal components.</u>

A. Comparison of 1000 Genome 2504 (all) samples with septic shock cohort samples.

B. Comparison of 1000 Genome 504 (East Asian population) sample with septic shock cohort samples.

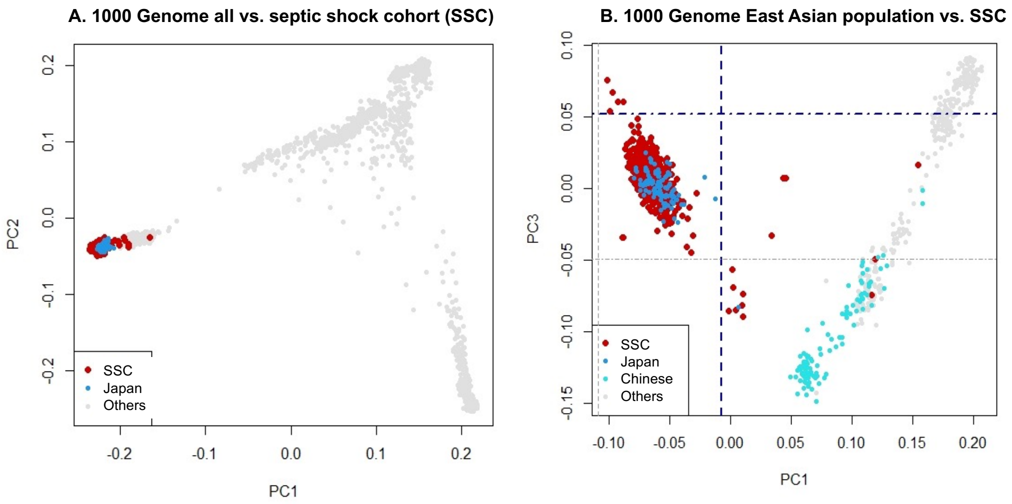

3. DNA Extraction and Genotyping

Genomic DNA was extracted from the buffy coat fraction of stored blood samples using the QIAamp DNA Mini Kit according to the manufacturer's instructions. Genotyping was performed using either the Illumina Human Omni or Illumina Infinium Omni microarray platform, which collectively genotyped approximately 2 million SNPs across the genome.

4. Two-Sample Mendelian Randomization Analysis

Rationale and Genetic Instrument Construction

To establish causal relationships independent of confounding and reverse causality, we performed a two-sample Mendelian randomization (MR) analysis. While polygenic score (PGS) approaches incorporate a broad spectrum of genetic variants, two-sample MR applies stringent selection criteria for genetic instruments, thereby providing stronger causal inference. Genetic instruments were constructed using summary statistics from the genome-wide association study (GWAS) employed to derive the testosterone PGS (GWAS Catalog accession number GCST90019520). Single nucleotide polymorphisms (SNPs) were selected as instrumental variables if they satisfied the following criteria: (1) genome-wide statistical significance ($P < 5 \times 10^{-8}$); (2) independence from other selected SNPs, with linkage disequilibrium $r^2 < 0.001$ within a 1 Mb clumping window; and (3) availability of summary statistics in the outcome dataset. These stringent criteria ensured instrumental variable validity and independence, minimizing bias from weak instruments and linkage disequilibrium. From the candidate SNPs, 53 were detectable in the septic shock cohort and retained as valid instruments for MR analysis.

For the outcome, we obtained summary statistics for 28-day mortality in septic shock patients from a previously published GWAS meta-analysis (as cited in the main manuscript).

Effect sizes for both exposure and outcome were standardized to per-standard deviation units to ensure comparability across datasets. During harmonization of exposure and outcome datasets, we carefully aligned effect alleles and excluded palindromic SNPs with intermediate allele frequencies to prevent strand ambiguity.

Causal Estimation Methods

The primary causal effect of testosterone on septic shock mortality was estimated using the inverse variance-weighted (IVW) method. The IVW method assumes that all genetic variants function as valid instrumental variables—that is, they are associated with serum testosterone levels (the exposure) but affect mortality (the outcome) only through their effect on

testosterone, with no direct effects on mortality or confounding pathways. The IVW method combines the variant-specific causal effect estimates (the ratio of effect on outcome to effect on exposure) weighted by the inverse of their variance, providing an overall estimate of causal association. Under this framework, the causal effect represents the change in mortality risk per unit increase in genetically predicted testosterone levels.

Pleiotropy Assessment and Sensitivity Analyses

Given that some genetic variants may violate the MR assumptions through pleiotropy (horizontal pleiotropy, where a single variant influences multiple biological pathways and affects mortality independent of testosterone), we employed complementary analytic approaches to strengthen causal inference:

1. Weighted Median Method: This approach provides consistent causal estimates if at least 50% of the weight in the combined analysis derives from valid instrumental variables. The weighted median is robust to the presence of invalid instruments and pleiotropy, making it valuable for sensitivity analysis.
2. MR-Egger Regression: This method assesses horizontal pleiotropy by examining whether the intercept term of the MR-Egger regression significantly deviates from zero. A non-zero intercept suggests directional pleiotropy, which could bias the IVW estimate. The MR-Egger slope provides a pleiotropy-robust causal estimate, though it requires the InSIDE (Instrument Strength Independent of Direct Effect) assumption.
3. MR-PRESSO (Pleiotropy Residual Sum and Outlier Detection): This method identifies genetic variants with outlier effect estimates that may exert disproportionate influence due to pleiotropy. MR-PRESSO performs both a global heterogeneity test and an outlier detection test; when significant outliers are detected, corrected causal estimates are recalculated after excluding these variants.
4. Leave-One-Out Sensitivity Analysis: We systematically excluded each instrumental variant in turn and recalculated the IVW causal estimate to assess whether any single SNP disproportionately influenced the overall result. This approach identifies potentially problematic variants and assesses the stability of the causal inference.

The consistency of causal estimates across these complementary methods and the absence of significant pleiotropy signals supported the robustness of our primary findings.

Table E1. Genome-wide significant variants for serum testosterone levels (n = 322,594).

Chr	Position (GRCh37)	SNP ID	Effect allele	Other allele	Beta	SE	*P* value
1	101740406	rs11166576	A	G	0.0362	0.0029	1.00E-36
1	107573565	rs2335077	G	A	0.0207	0.0026	2.65E-15
1	113098534	rs6658555	T	C	-0.0181	0.0029	4.19E-10
1	155940948	rs11264426	G	T	0.0177	0.0029	6.51E-10
1	179258507	rs2257089	G	A	0.0268	0.0029	1.30E-20
1	179319816	rs12140800	G	A	-0.0156	0.0026	2.40E-09
1	218542059	rs6662137	G	T	-0.0164	0.0027	8.58E-10
2	27730940	rs1260326	C	T	0.044	0.0026	1.73E-65
2	32183611	rs7559329	C	T	0.0248	0.0035	6.54E-13
2	43506259	rs7561670	A	G	-0.021	0.0032	3.26E-11
2	111988016	rs6749633	A	G	0.0237	0.0028	1.23E-17
2	165513091	rs10195252	C	T	0.0153	0.0026	2.20E-09
2	234621825	rs3755321	C	T	-0.0365	0.0044	2.25E-16
4	3473139	rs6831256	G	A	0.0147	0.0025	5.99E-09
4	69961127	rs11940316	C	T	-0.0245	0.0025	2.71E-22
4	88064431	rs10023050	G	A	0.015	0.0026	4.69E-09
4	106083776	rs2285720	T	C	0.0165	0.0026	2.66E-10
5	55861894	rs9687846	A	G	-0.0231	0.0031	1.13E-13
5	76483059	rs73767325	A	G	-0.0152	0.0026	4.69E-09
6	30912481	rs2844702	A	G	0.0188	0.0026	5.95E-13
6	31603591	rs2261033	G	A	0.0191	0.0025	3.94E-14
6	43355851	rs2396004	G	A	0.0234	0.0025	2.01E-20
6	105378954	rs7759938	T	C	0.0171	0.0027	2.12E-10
6	119128633	rs415441	T	C	0.0299	0.0029	3.27E-24
7	15026516	rs10257710	A	C	-0.0177	0.0025	1.59E-12
7	73012042	rs35332062	A	G	0.0293	0.0037	5.26E-15
7	98005398	rs2107717	C	T	0.017	0.0027	2.39E-10
7	99081730	rs6962772	G	A	-0.0411	0.0035	1.82E-32
7	99494030	rs2572005	A	G	0.015	0.0025	4.22E-09
7	137801915	rs7780066	A	G	0.0274	0.0031	8.08E-19
8	105959223	rs5893709	C	CT	0.0213	0.0032	3.04E-11
8	143957856	rs7822986	A	G	-0.0213	0.0025	2.15E-17
9	114812743	rs1864357	A	G	-0.0207	0.0031	3.56E-11
10	5055950	rs7099721	G	A	-0.0173	0.0026	5.81E-11
10	64761387	rs72820803	T	C	-0.0315	0.0027	1.17E-31
10	104623578	rs9527	T	C	-0.0257	0.003	4.28E-18
11	72327205	rs3781931	T	C	-0.0223	0.0035	1.34E-10
11	122736571	rs10892919	T	C	0.0164	0.0025	9.44E-11
13	22321690	rs544400	T	C	-0.0236	0.0025	4.98E-21
13	22340878	rs58928587	A	G	-0.017	0.0026	8.46E-11
15	40360741	rs7181230	G	A	0.0256	0.0026	1.55E-22
15	51519276	rs4775935	G	T	-0.0191	0.0026	3.13E-13
15	79856167	rs8033269	C	T	-0.0208	0.0035	2.44E-09
15	96708291	rs8023580	C	T	0.0299	0.0028	9.90E-27
16	81570478	rs9928591	T	C	0.048	0.0053	2.59E-19
17	7321858	rs11078677	T	C	0.0479	0.0028	7.07E-68

17	7491177	rs4227	T	G	-0.0876	0.0028	2.42E-217
17	7613765	rs3744262	A	G	0.0435	0.0027	3.11E-58
17	47314413	rs624177	G	A	0.0163	0.0026	5.34E-10
17	47448172	rs11655704	C	T	0.0268	0.0027	1.21E-23
18	71938536	rs17089028	A	G	-0.0375	0.0037	1.93E-24
19	12505873	rs7246440	G	A	-0.0189	0.0029	8.82E-11
19	41985587	rs11672691	G	A	-0.019	0.0029	4.05E-11

Chr, Chromosome; SE, standard error

Table E2. Brussels organ dysfunction definitions.

			Clinically Significant Organ Dysfunction		
Organs	Normal	Mild	Moderate	Severe	Extreme
Cardiovascular (systolic blood pressure, mmHg)	>90	≤ 90 Fluid responsive	≤ 90 not fluid responsive	≤ 90 pH 7.3	≤ 90 pH 7.2
Pulmonary (PaO_2/FIO_2, mmHg)	>400	301-400	201-300	101-200	100
Neurologic (GCS)	15	13-14	10-12	6-9	≤ 5
Coagulation (platelet count, $x10^3/mm^3$)	>120	81-120	51-80	21-50	≤20
Renal (creatinine, µmol/L)	<133	133-175	176-300	301-442	≥443
Hepatic (bilirubin, µmol/L)	<20	20-32	33-99	100-199	≥200

Days alive and free (DAF) calculations. DAF was scored as 1 if the patient was alive and free of organ dysfunction (normal or mild dysfunction). DAF was scored as 0 if the patient had organ dysfunction (moderate, severe, or extreme) or was not alive. Each of the 28 days after meeting the inclusion criteria was scored. For any 24-hour period in which there is no measurement of a variable, we carried forward the value from the previous 24-hour period. If a variable was never measured, it was assumed to be normal. Once a patient was discharged home, they were considered free of organ failure.

Figure E1. Adjusted models for genetically predicted testosterone levels and 28-day mortality.

We assigned each patient a genetically predicted testosterone level using a polygenic score and then tested the association with 28-day mortality using a Cox proportional model adjusted for age, sex, steroid use, and BMI.

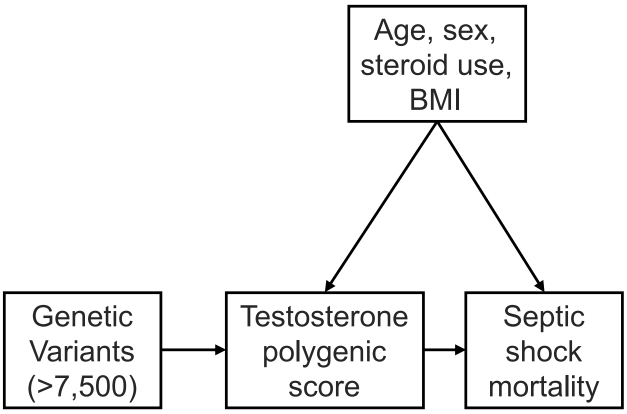

Figure E2. The effect of each SNP on Mendelian randomization analysis.

Leave-one-out analysis was performed to explore SNPs influencing the results on Mendelian randomization.

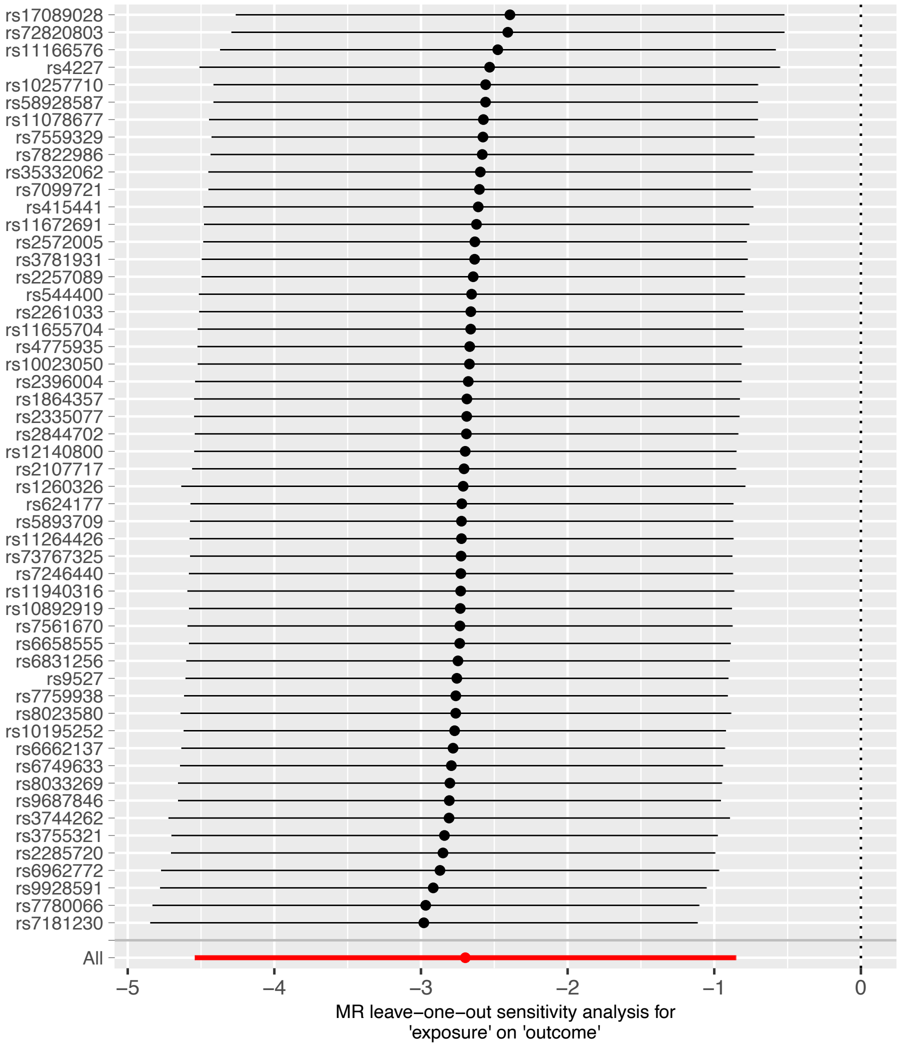

Figure E3. Association of genetically predicted testosterone levels with lipoproteins measured during septic shock.
The association was analyzed by linear regression: apolipoprotein C3 levels were significantly positively correlated with genetically predicted testosterone levels, but none of the correlations were significant after correction for multiple testing (Bonferroni correction).

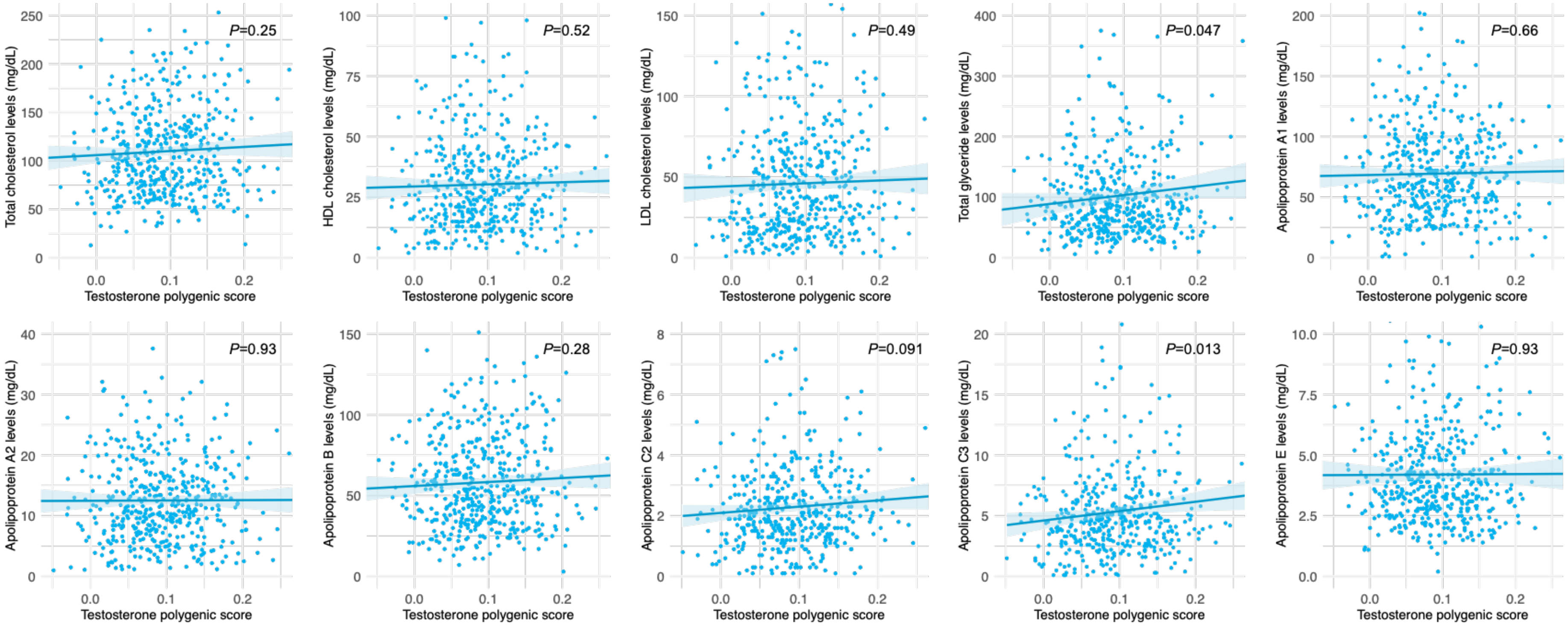

Supplement: Supplementary file 1 — Supplementary Material 1 [file 13054_2026_5860_MOESM1_ESM.pdf]
